# Did COVID-19 or COVID-19 Vaccines Influence the Patterns of Dengue in 2021? An Exploratory Analysis of Two Observational Studies from North India

**DOI:** 10.4269/ajtmh.23-0418

**Published:** 2023-10-30

**Authors:** Upinder Kaur, Parth Jethwani, Shraddha Mishra, Amol Dehade, Ashish Kumar Yadav, Sasanka Chakrabarti, Sankha Shubhra Chakrabarti

**Affiliations:** ^1^ Department of Pharmacology, Institute of Medical Sciences, Banaras Hindu University, Varanasi, India;; ^2^ Department of Endocrinology and Metabolism, All India Institute of Medical Sciences, Jodhpur, India;; ^3^ Center for Biostatistics, Institute of Medical Sciences, Banaras Hindu University, Varanasi, India;; ^4^ Department of Biochemistry, Maharishi Markandeshwar (deemed to be University), Mullana, India;; ^5^ Central Research Cell, Maharishi Markandeshwar (deemed to be University), Mullana, India;; ^6^ Department of Geriatric Medicine, Institute of Medical Sciences, Banaras Hindu University, Varanasi, India

## Abstract

Dengue experienced a rise in disease burden in 2021 in specific regions of India. We aimed to explore the risk factors of dengue occurrence and severity in the post-COVID-19 and post-COVID-19 vaccination era and performed an exploratory analysis involving participants from two prior observational studies conducted from February 2021 to April 2022 in a tertiary hospital in North India. Health care workers constituted the majority of the study participants. Individuals were stratified into five groups based on COVID-19 infection and timing of vaccination: COVID-No Vaccine, Vaccine-No COVID (VNC), COVID After Vaccine (CAV), Vaccine After COVID (VAC), and No Vaccine-No COVID (NVNC) groups. The occurrence of laboratory-confirmed dengue and severe forms of dengue were the main outcomes of interest. A total of 1,701 participants (1,520 vaccinated, 181 unvaccinated) were included. Of these, symptomatic dengue occurred in 133 (7.8%) and was “severe” in 42 (31.6%) cases. Individuals with a history of COVID-19 in 2020 had a 2-times-higher odds of developing symptomatic dengue (*P =* 0.002). The VAC group had 3.6 (*P =* 0.019)-, 2 (*P =* 0.002)-, and 1.9 (*P =* 0.01)-times-higher odds of developing symptomatic dengue than the NVNC, VNC, and CAV groups, respectively. The severity of dengue was not affected by COVID-19 vaccination but with marginal statistical significance, a 2-times-higher risk of severe dengue was observed with any COVID-19 of the past (*P =* 0.08). We conclude that COVID-19 may enhance the risk of developing symptomatic dengue. Future research should explore the predisposition of COVID-19-recovered patients toward other viral illnesses. Individuals receiving COVID-19 vaccines after recovering from COVID-19 particularly seem to be at greater risk of symptomatic dengue and need long-term watchfulness. Possible mechanisms, such as antibody-dependent enhancement or T-cell dysfunction, should be investigated in COVID-19-recovered and vaccinated individuals.

## INTRODUCTION

COVID-19 has affected more than 0.75 billion people globally and claimed nearly 7 million lives.^1^ In the fight against the virus, COVID-19 vaccination programs were started across the world in late 2020. In India, the mass rollout of COVID-19 vaccines was initiated in January 2021, with the ChAdOx1-nCoV-19 vaccine (COVISHIELD, Serum Institute of India under license from Oxford-AstraZeneca) and the inactivated severe acute respiratory syndrome coronavirus 2 (SARS-CoV-2) vaccine BBV152 (COVAXIN, Bharat Biotech) primarily employed. The country, however, was hit by SARS-CoV-2 during the second wave of the pandemic, which started in the middle of March 2021 and peaked in the first week of May 2021 with daily confirmed cases of COVID-19 reaching as high as 0.38 million.^2^ In this regard, we previously reported a high rate (25–40%) of breakthrough infections in the ChAdOx1-nCoV-19-vaccinated cohort composed primarily of health care workers at the Institute of Medical Sciences.^3^ The virulent delta strain of SARS-CoV-2 was considered responsible for the devastating second wave of the COVID-19 pandemic.^4^ In the following months, from July to September 2021, coinciding with seasonal trends, a surge of dengue cases was observed in the country with the reported burden and deaths being more than 4 and 6 times higher, respectively than those of the year 2020.^5^ In Uttar Pradesh, the numbers were even more substantial, with the reported number of cases more than 8 times higher than that of 2020.^5^ We also witnessed a similarly high rate of infections and hospital admissions because of dengue in health care workers of our institute, a pattern that was not observed over the last 5 years. The reasons and determinants of these disturbing trends of occurrence and severity of dengue have not been adequately studied. The possible modulation of patterns of dengue by COVID-19 and COVID-19 vaccination has been unexplored. The present study aims to determine the risk factors of dengue occurrence and severity in the post-COVID-19 and post-COVID-19 vaccination era. Particularly, we have tried to explore the effect of COVID-19 and COVID-19 vaccination on symptomatic dengue occurrence and dengue severity.

## MATERIALS AND METHODS

### Study design and participants.

The present study is an exploratory analysis and involved participants from two studies conducted in the Sir Sunder Lal Hospital, which is a large tertiary care referral center in northern India. The first study was a prospective observational safety study of individuals vaccinated with ChAdOx1-nCoV-19 and was conducted for the period of February 2021 to April 2022. The safety of ChAdOx1-nCoV-19 and the occurrence of COVID-19 after vaccination were the main outcomes of the study, and several findings have been published by us.^3^^,^^6^ Being the priority vaccine recipients in the first phase of COVID-19 vaccination, health care workers and the elderly constituted the study population. In view of the observed variations in patterns of dengue at our institute, dengue was incorporated as a separate entity in the list of adverse events of special interest (AESIs) in the final follow-up of this 1-year-long safety study.^7^ The severity of dengue as an AESI was defined in accordance with the Food and Drug Administration’s (FDA) rating of adverse events after immunization. Any form of dengue that required intravenous fluid therapy at home (FDA grade “severe”) or resulted in hospitalization (FDA grade “serious”) with or without the need for platelet transfusion was grouped under “severe” dengue and considered as dengue of significant severity. To make meaningful comparisons of the vaccinated cohort from this study with an unvaccinated cohort with similar demographic and occupational risk factors, we drew unvaccinated participants from a second study whose findings were also published by us previously. This second study was based on a retrospective cohort design with the primary objective of determining COVID-19 vaccine effectiveness and thus enrolled both vaccinated and unvaccinated health care workers of our institute.^8^ This study was conducted from July 2021 to December 2021. The unvaccinated individuals from this study were contacted again during the months of October to November 2022 to obtain the dengue-related information of the year 2021. Vaccination status was again confirmed for these individuals, as it was possible that they had received COVID-19 vaccines in the intervening period. For the present analysis, unvaccinated individuals were defined as those who had not taken any dose of the COVID-19 vaccine until the major curve of dengue cases touched the baseline (until September 30, 2021 based on data collected from the study participants), termed hereafter the “end date.” All those who had taken at least one dose of the COVID-19 vaccine before July 1, 2021, which coincided with the start of the dengue epidemic (termed hereafter the “start date”), were grouped together in the vaccinated arm. To prevent adulteration by cases who received vaccines during the dengue epidemic period (July 1, 2021 to September 30, 2021), individuals receiving their first dose of vaccine during this period were excluded from the present analysis.

To understand the cumulative effect of COVID-19 occurring at any time on dengue occurrence and severity and to explore the effect of the timing of the COVID-19 vaccine with respect to COVID-19 illness on dengue occurrence and severity, participants were categorized into five groups as defined below.

### Categorization A.

1. COVID-No Vaccine (CNV) group: Individuals who had a history of COVID-19 before the “start date” but were unvaccinated until the “end date.”
2. Vaccine-No COVID (VNC) group: Individuals who had received at least one dose of COVID-19 vaccine before the “start date” but had no history of COVID-19 till the “start date.”
3. COVID After Vaccine (CAV) group: Individuals who, after receiving the COVID-19 vaccine, developed COVID-19 at any time but before the “start date.”
4. Vaccine After COVID (VAC) group: Individuals who had a history of COVID-19 in the year 2020 and then received COVID-19 vaccine in the year 2021 at any time but before the “start date.”
5. No Vaccine-No COVID (NVNC) group: Individuals who had no history of COVID-19 till the “start date” and were unvaccinated till the “end date.”

### Categorization B.

As mentioned above, our institute was hit by the second wave of the COVID-19 pandemic in the months of March to May 2021. Also, as per the revised recommendations by the authorities on COVID-19 vaccination, the interval between the two doses of the COVID-19 vaccine was extended to 84 days. Consequently, some participants received their second dose of the COVID-19 vaccine after recovery from COVID-19 of the year 2021. The individuals who had received their second dose of the COVID-19 vaccine after COVID-19 of the year 2021, but before the development of dengue, were shifted from the CAV group of categorization A to the VAC group under categorization B and analyzed separately.

### Ethical concerns.

Ethical permission was obtained from the Institute Ethics Committee, and informed consent was taken from the study participants.

### Outcomes.

The rate of occurrence of laboratory-confirmed symptomatic dengue and the rate of severe forms of dengue were the main outcomes studied in the present analysis. In addition, we predicted the determinants of dengue occurrence and severity, with a particular focus on COVID-19 history and vaccination status.

### Statistical analysis.

The χ^2^ test was used to assess the association between dichotomous variables such as the outcome variables pertaining to dengue and independent variables such as demographics, comorbidities, COVID-19, and COVID-19 vaccination status. The variables with a *P* < 0.05 in unadjusted bivariate analysis and those considered to be clinically relevant were incorporated in the adjusted logistic regression analysis, with dengue-specific outcome measures (symptomatic dengue occurrence, severity) being the dependent variables. An interaction variable was included in the model for variables with a *P* < 0.05 in unadjusted analysis. To check the model specifications, a link test was performed using Stata version 16 (StataCorp LLC, College Station, TX). The model adequacy and diagnostics were checked using the log-likelihood test, Akaike information criterion (AIC), and Bayesian information criterion (BIC) values. The goodness of fit was graphically assessed using the standardized deviance residuals (DRi) and standardized Pearsonian residuals. A separate bivariate analysis was conducted to find the association between the timing of the COVID-19 vaccine with respect to COVID-19 and the occurrence of symptomatic dengue/severe dengue, and this was followed by a logistic regression analysis for the same.

## RESULTS

**Figure 1.**
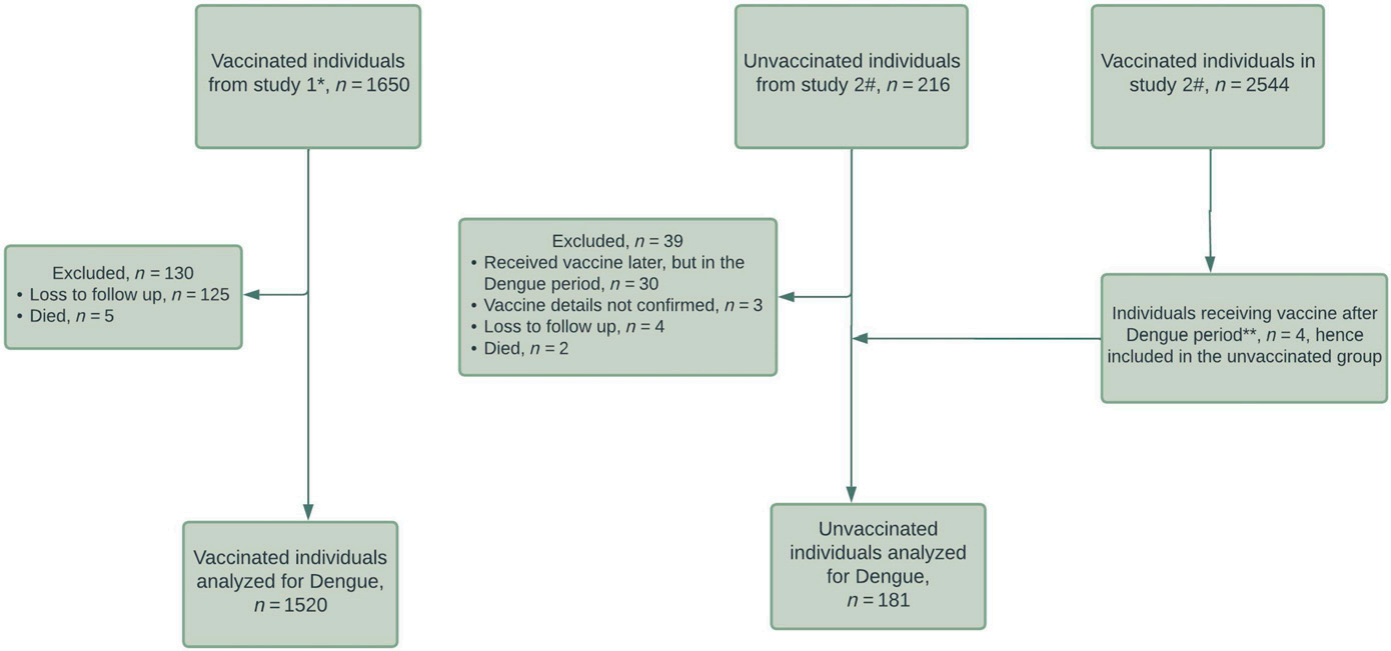
STROBE flowchart of enrollment of study participants. *Reference 6; #Reference 8; **dengue epidemic period is considered to be from July 1, 2021 to September 30, 2021.

Figure 1 shows the flowchart of study participants. Out of 1,650 participants from study 1 who were vaccinated with the ChAdOx1-nCoV-19 vaccine, 1,520 could be contacted for dengue analysis. As mentioned earlier, health care workers of our institute dominated the group. From study 2 conducted in the same institute, out of 216 unvaccinated health care workers, 181 were included in the present analysis.

### Estimates of dengue and its severity.

Table 1 shows the distribution of demographic characteristics, comorbidities, history of COVID-19, and COVID-19 vaccination status with respect to the occurrence of symptomatic dengue. Out of a total of 1,701 participants, COVID-19 occurred in 231 (13.6%) participants during the year 2020 and in 579 (34%) individuals during the year 2021, before the occurrence of dengue in the defined dengue epidemic period. Symptomatic dengue was reported in 133 (7.8%) individuals. Among dengue cases, “severe” dengue occurred in 42 (31.6%) participants. A significant association for the occurrence of symptomatic dengue was observed with age and COVID-19 of the first wave (i.e., 2020). Symptomatic dengue occurred commonly in individuals with a history of COVID-19 in the first wave and in young individuals < 40 years of age. No association existed, however, with COVID-19 of the second wave or vaccination status. Table 1 also shows the association of the severity of dengue with the same variables. The severity of dengue did not share a significant association with any of the variables.

**Table 1:** Association of occurrence of dengue and severity of dengue with demographics, comorbidities, COVID-19, and COVID-19 vaccination

Parameter	*N* = 1,701	Dengue cases, *n* (%)*	*P* value	RR (CI)	*n* = 133	Severe dengue, *n* (%)†	*P* value	RR (CI)
Age (years)								
< 40	1,104	97 (8.8)	**0.04**	**1.46** (1.01–2.10)	97	32 (33)	0.56	–
≥ 40	597	36 (6)		–	36	10 (27.8)		–
Sex								
Female	563	44 (7.8)	0.99	–	44	17 (38.6)	0.22	–
Male	1,138	89 (7.8)		–	89	25 (28.1)		–
BMI‡								
< 25	948	77 (8.1)	0.60	–	77	26 (33.8)	0.52	–
≥ 25	752	56 (7.4)		–	56	16 (28.6)		–
COVID-19 in year 2020								
No	1,470	102 (6.9)	**0.001**	–	102	32 (31.4)	0.93	–
Yes	231	31 (13.4)		**1.93** (1.32–2.81)	31	10 (32.3)		–
COVID-19 in year 2021								
No	1,122	82 (7.3)	0.27	–	82	23 (28)	0.27	–
Yes	579	51 (8.8)		–	51	19 (37.3)		–
Any COVID-19 before dengue								
No	961	63 (6.6)	**0.027**	–	63	15 (23.8)	0.07	–
Yes	740	70 (9.5)		**1.44** (1.04–2.0)	70	27 (38.6)		1.62 (0.95–2.75)
Diabetes mellitus								
No	1,561	123 (7.9)	0.76	–	123	37 (30.1)	0.28	–
Yes	140	10 (7.1)		–	10	5 (50)		–
Hypertension								
No	1,525	119 (7.8)	0.94	–	119	39 (32.8)	0.55	–
Yes	176	14 (8.1)		–	14	3 (21.4)		–
Heart disease								
No	1,674	130 (7.8)	0.46	–	130	41 (31.5)	1.0	–
Yes	27	3 (11.1)		–	3	1 (33.5)		–
Lung disease								
No	1,653	128 (7.7)	0.42	–	128	42 (32.8)	0.18	–
Yes	48	5 (10.4)		–	5	0		–
Hypothyroidism								
No	1,637	128 (7.8)	0.99	–	128	41 (32)	1.0	–
Yes	64	5		–	5	1 (20)		–
Vaccination status								
Vaccinated	1,520	122 (8)	0.36	–	122	37 (30.3)	0.32	–
Unvaccinated	181	11 (6.1)		–	11	5 (45.5)		–

BMI = body mass index. Bold values denote statistically significant findings.

* Percentage calculated out of total participants analyzed (*N* = 1,701).

† Percentage calculated out of dengue cases (*n* = 133).

‡ Not known for one participant.

### Risk factors of dengue occurrence and severity.

From the results of unadjusted bivariate analysis (Table 1), a history of COVID-19 in the year 2020 and age shared a statistically significant association with occurrence of symptomatic dengue. Because COVID-19 of the year 2021 and vaccination status might have also impacted the dengue estimates, which the study aimed to explore, these two were also included in the regression model. Table 2 shows the risk factors for the occurrence of symptomatic dengue. COVID-19 in the year 2020 was an independent risk factor of symptomatic dengue (adjusted odds ratio [aOR] = 2, *P =* 0.002). No risk of development of dengue was noticed with COVID-19 of the year 2021, i.e., the second wave of COVID-19, or with COVID-19 vaccination. The model diagnostics are described in the footnote of Table 2. The interaction between COVID-19 of the year 2020 and age was also studied and was not found to be significant in the model (*P =* 0.64). The severity of dengue was not influenced by COVID-19 vaccination. However, with marginal statistical significance, a 2-times-higher risk of “severe” dengue was noticed with a history of any COVID-19 in the past, as shown in Table 2.

**Table 2:** Tentative risk factors of occurrence and severity of dengue in adjusted analysis (logistic regression)

Risk factor	aOR (CI)	*P* value
Risk factors of occurrence of dengue, *N* = 1,701		
Age (years)		
< 40	1.44 (0.96–2.16)	0.07
≥ 40	Reference	
COVID-19 in year 2020		
Yes	**2** (1.30–3.09)	**0.002**
No	Reference	
COVID-19 in year 2021		
Yes	1.2 (0.84–1.75)	0.31
No	Reference	
Vaccination status		
Unvaccinated	0.69 (0.36–1.32)	0.27
Vaccinated	Reference	
Risk factors of severity of dengue, *N* = 133		
Any COVID-19		
Yes	2.0 (0.93–4.17)	0.08
No	Reference	
Vaccination status		
Unvaccinated	1.8 (0.5–6.3)	0.36
Vaccinated	Reference	

aOR = adjusted odds ratio.

Model diagnostics for dengue occurrence: omnibus test of model coefficient, *P* = 0.004; B, −2.46; Wald, 746.28; Hosmer-Lemeshow test, *P* = 0.67; Link test, *P* = 0.39. The Akaike information criterion value was 927.62 by inclusion of all four variables as described above and 925.8 if only two variables (COVID-19 of the year 2020 and age) were considered in the model, making the present model a good fit. No pattern of standardized deviance residuals was observed in the index plot. The interaction between COVID-19 of the year 2020 and age was not statistically significant (*P* = 0.64). Bold values denote statistically significant findings.

### Risk of dengue based on timing of vaccine with respect to COVID-19.

**Figure 2.**
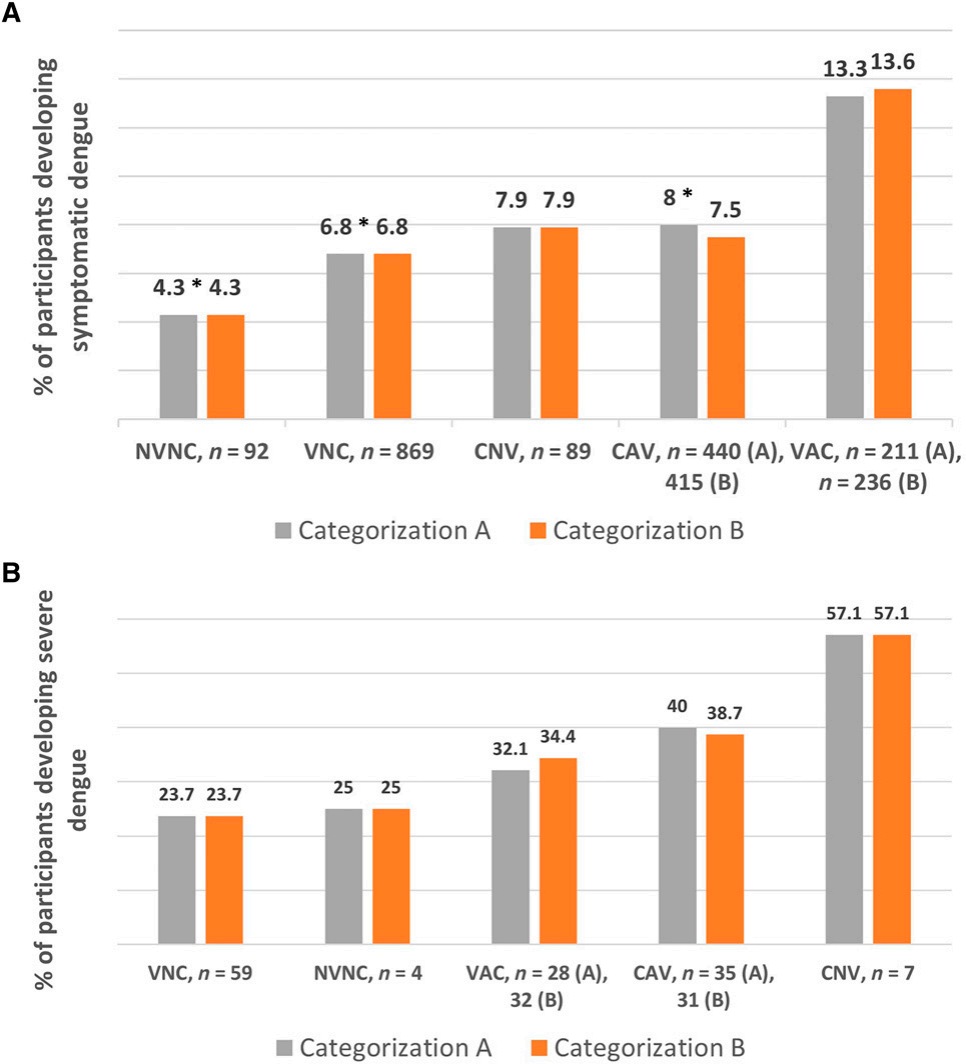
(**A**) Occurrence rate of dengue in study participants categorized based on COVID-19 illness at any time (before dengue), vaccination status, and timing of COVID-19 vaccine with respect to COVID-19 illness (as per categorizations A and B). The number of individuals belonging to each group is displayed on the horizontal axis along with the group label. The numbers differed in CAV and VAC groups as per categorizations A and B. *P value < 0.05 compared with VAC. (**B**) Severity of dengue in study participants categorized based on COVID-19 illness at any time (before dengue), vaccination status, and timing of COVID-19 vaccine with respect to COVID-19 illness (as per categorizations A and B). The number of individuals belonging to each group is displayed on the horizontal axis along with the group label. The numbers differed in CAV and VAC groups as per categorizations A and B. CAV = COVID After Vaccine; CNV = COVID-No Vaccine; NVNC = No Vaccine-No COVID; VAC = Vaccine After Covid; VNC = Vaccine-No COVID.

Figure 2A and B reflect the occurrence and severity of dengue when participants were classified based on their prior history of COVID-19, vaccination status, and timing of the vaccine with respect to COVID-19 of the year 2020 or 2021. The number of participants belonging to each group is mentioned in the figures. Twenty-five participants of this CAV group subsequently received their second dose of vaccine after COVID-19 of the year 2021 and hence were moved to the VAC group of categorization B for further sensitivity analysis. With statistical significance (*P =* 0.02), the highest occurrence (13.3%) of dengue was noticed in individuals of the VAC group of categorization A. The rates were low in the CAV (8%), CNV (7.9%), and VNC (6.8%) groups. The NVNC group had the lowest rates of dengue (4.3%). Similar patterns of incidence of dengue were observed in individuals classified as per categorization B (Figure 2A). No statistically significant difference was observed between these groups with respect to dengue severity analyzed by categorization A (*P =* 0.26) or categorization B (*P =* 0.28) (Figure 2B). Group-specific risk estimates of dengue severity were calculated by both categorizations, and odds ratios are mentioned in Supplemental Table 1. No increased risk of dengue severity was observed in any group by either categorization.

Table 3 shows the regression analysis results of occurrence of symptomatic dengue according to group categorization. Compared with individuals with no history of COVID-19 and no history of COVID-19 vaccination (NVNC group), a 3.5-times-higher odds of dengue occurrence was observed in individuals receiving COVID-19 vaccine after recovery from prior COVID-19 of the year 2020 (VAC group) (*P =* 0.023). In addition, the VAC group had 1.98-times-higher and 1.74-times-higher odds of dengue than individuals of the VNC group and those of the CAV group, respectively. Results were corroborated in another analysis as per categorization B (Table 3). A 3.6-, 2.04-, and 1.92-times-higher odds of dengue was observed in the VAC group compared to the NVNC, VNC, and CAV groups, respectively.

**Table 3:** Risk factors of occurrence of dengue depending on categorization of participants based on COVID-19 at any time, vaccination status, and timing of vaccine with respect to COVID-19 episode

Risk factor	VAC includes individuals with history of receiving vaccine in 2021 after COVID-19 of the year 2020		VAC includes individuals with history of receiving COVID-19 vaccine in 2021 after COVID-19 of the year 2020 as well as individuals receiving second dose of COVID-19 vaccine after COVID-19 of the year 2021 but before development of dengue	
	aOR (CI)	*P* value	aOR (CI)	*P* value
Age (years)				
< 40	1.46 (0.97–2.2)	0.065	1.46 (0.98–2.2)	0.06
≥ 40 (Reference)	–	–	–	–
Categories of participants				
NVNC (reference)				
VAC	**3.5** (1.2–10.2)	**0.023**	**3.6** (1.2–10.5)	**0.019**
CNV	1.85 (0.52–6.5)	0.34	1.85 (0.52–6.5)	0.34
VNC	1.7 (0.62–4.9)	0.29	1.75 (0.62–4.96)	0.29
CAV	2 (0.69–5.8)	0.19	1.87 (0.64–5.4)	0.25
VNC (reference)				
VAC	**1.98** (1.23–3.20)	**0.005**	**2.04** (1.3–3.2)	**0.002**
CAV (reference)				
VAC	**1.74** (1.02–2.9)	**0.04**	**1.92** (1.14–3.23)	**0.015**
CNV (reference)				
VAC	1.8 (0.79–4.5)	0.15	1.94 (0.82–4.58)	0.13

aOR = adjusted odds ratio; CAV = COVID After Vaccine; CNV = COVID-No Vaccine; NVNC = No Vaccine-No COVID; VAC = Vaccine After COVID; VNC = Vaccine-No COVID. Bold values denote statistically significant findings.

## DISCUSSION

With about 70% of the disease burden from Asia, dengue continues to remain endemic in more than 100 countries.^9^ In India, the disease burden has seen an unprecedented rise in recent times, posing substantial challenges to the health infrastructure.^5^ The dengue virus (DENV) belongs to the *Flavivirus* genus and has four serotypes (DEN1, DEN2, DEN3, and DEN4), primarily caused by the bite of infected *Aedes aegypti* mosquitoes. DEN2 is the most prevalent serotype in India, and although recovery from infection provides immunity against a particular serotype, secondary infection by other serotypes continues to remain a challenge, particularly in hyperendemic regions.^10^ As per the CDC, one out of four infected cases is symptomatic, symptoms being governed by the immune responsiveness of the host and the level of viremia.^11^^,^^12^

The institute where the present study was conducted faced the brunt of the second wave of COVID-19 during the months of March to May 2021 and dengue during the period of July to September 2021. The present study was hence planned to explore a possible relationship between COVID-19, COVID-19 vaccines, and dengue. The occurrence of dengue was two times more common in individuals with a history of COVID-19 in the first wave of the pandemic, i.e., in the year 2020. There was a trend towards a higher risk of occurrence of dengue in individuals less than 40 years of age. Interestingly, the occurrence of dengue was not determined by COVID-19 of the second wave nor was it affected by the vaccination status of participants. However, a significant association was observed when, apart from vaccination status and history of COVID-19, individuals were categorized depending upon their cumulative COVID-19 and vaccination status and timing of vaccination with respect to COVID-19. Individuals who received the vaccine after recovery from COVID-19 had a 3.6-times-higher risk of developing dengue in 2021 than individuals with no history of COVID-19 illness and vaccination. This same group was at higher risk of developing dengue than vaccinated individuals with no history of COVID-19 and individuals developing COVID-19 after receipt of the vaccine. The timing of the COVID-19 vaccine has recently been linked with the persistence of long COVID symptoms also. Vaccines received after recovery from prior COVID-19 have been linked to persistent long-term COVID symptoms in comparison with vaccines received before COVID-19 disease.^8^

With respect to the severity of dengue, no statistically significant association was observed with COVID-19 or COVID-19 vaccines. However, with marginal statistical significance, a 2-times-higher risk of severe dengue was noticed in individuals with any history of COVID-19 before dengue. Also, a numerically higher rate of “severe” dengue was reported in unvaccinated individuals with a history of COVID-19 before dengue. However, relevant conclusions cannot be generated because of the small number of unvaccinated people overall and the meager representation of severe dengue in the study. Recently, convalescent sera of patients diagnosed with COVID-19 in 2020 were shown to neutralize dengue virus in in vitro studies and to limit the progression of severe dengue in animal models.^13^^,^^14^ Few clinical studies have assessed the association between the seroprevalence of COVID-19 antibodies and dengue severity in children, and the results have been discordant.^15^^,^^16^ In light of the discrepancy in preclinical and clinical studies, the findings of the present study warrant validation from larger epidemiological studies.

The present study assessed the severity of dengue in terms of intravenous fluid requirement or need for hospitalization. Two study participants required platelet transfusion. Of these, one had developed COVID-19 in the second wave of the pandemic after being fully vaccinated and later developed severe dengue with a nadir platelet count of 10,000/µL. The other had a history of COVID-19 during the second wave but was unvaccinated until the date of collection of data and developed severe dengue with a platelet count as low as 8,000/µL. Mucosal bleeding in the form of epistaxis with moderate thrombocytopenia occurred in one participant who was fully vaccinated but had no history of COVID-19.

The underlying mechanisms by which COVID-19 modulates the occurrence and severity of dengue have not been fully elucidated. A mechanistic understanding of chronic immunological perturbations by SARS-CoV-2 will lend more insights into how SARS-CoV-2 may potentially enhance the vulnerability of an individual to symptomatic dengue. Recently, a decline in CD8^+^ T cells and CD4^+^ T cells and an increase in CD16^+^ monocytes has been observed in COVID-19 convalescent patients even after 6 to 8 months of recovery from the infection.^17^ Previously, a low frequency of gamma interferon (IFN-γ)- and interleukin 2 (IL-2)-producing T cells has been linked with clinically manifested forms of dengue, whereas a high frequency of corresponding cells conferred protection from symptomatic dengue.^18^ CD4^+^ and CD8^+^ T cells curtail the infection by the production of IFN-γ, tumor necrosis factor alpha, and IL-2 as well as by direct cytotoxicity or lysis of infected cells.^19^ Accordingly, the persistent lymphopenia observed in the post-COVID-19 state may result in uncontrolled replication of dengue virus inside host cells. The higher rates of symptomatic dengue in individuals with a history of COVID-19 and specifically in individuals receiving COVID-19 vaccine after natural infection might also be explained by the phenomenon of antibody-dependent enhancement (ADE) of infection. The ADE in dengue occurs when a person after recovery from a primary infection by one serotype suffers from a secondary heterotypic infection with another serotype. The cross-reactive antibodies produced by primary infection fail to neutralize the secondary infection and instead form complexes with the new strain. The resulting interaction of the virus with Fcγ receptors of monocytes promotes viral entry into host cells and facilitates viral replication.^20^ Classically, ADE triggers severe forms of dengue such as dengue hemorrhagic fever and dengue shock syndrome by producing an aberrant “cytokine storm.” Theoretically, however, ADE decides the level of viremia and hence can also influence the patterns of presentation of dengue, whether subclinical or symptomatic. Worth investigating in continuation is whether the anti-S or anti-receptor-binding domain (RBD) antibodies produced in response to natural SARS-CoV-2 infection or vaccination may facilitate the disease course of dengue. Recently, the anti-S-RBD antibodies produced in response to SARS-CoV-2 infection have been shown to recognize the envelope protein and NS1 protein of DENV.^13^^,^^14^ To what extent and in which direction the cross-reactivity between SARS-CoV-2 and dengue can modulate the disease course of either is also worth exploring. Although DENV serotyping was not done in the study participants, ADE has also been projected to produce cyclical and chaotic epidemics with enhanced strains in various mathematical models of dengue dynamics.^21^

The concept of cross-reactive immune response between different viruses gains support from reports of outbreaks of Zika virus in dengue-endemic regions of America. Antibodies against structural proteins of DENV cross-react with structural epitopes of the Zika virus. The subsequent interaction has been shown to produce minimal to enhancing effects on Zika infection and pathogenesis.^22^^,^^23^ Antibodies against Zika virus also show cross-reactivity with DENV, and enhanced dengue occurrence and severity have been noticed in dengue-prone animal models upon administration of Zika-specific antibodies.^24^ The cellular response mounted against DENV or Zika virus, on the other hand, is mutually cross-protective. The predominant humoral response generated by DENGVAXIA (Sanofi Pasteur, Inc.), a yellow fever virus-based dengue vaccine against the structural protein of DENV, has been viewed as a reason for a nonsatisfactory protection against dengue. Accordingly, vaccines based on nonstructural proteins of DENV can rather impart a stronger protection against dengue and possibly against Zika. Vaccination platforms used against COVID-19 are based primarily on the mounting of the humoral response against the structural spike protein of SARS-CoV-2. A rather high vulnerability of vaccinated individuals to the original strain as well as variants of SARS-CoV-2 has been observed in the early months of the postvaccination period.^8^^,^^25^^,^^26^ Along these lines, a vaccine also based on the nonstructural proteins of SARS-CoV-2 with the generation of a protective T-cell response is a requisite in the fight against SARS-CoV-2, its variants, and possibly other viruses with similarity in epitopes.

### Limitations.

A major drawback of the present study is the small sample size of the unvaccinated arm. Larger population-level studies with a sufficient number of unvaccinated people are needed to understand the relationship between COVID-19, the COVID-19 vaccines, and dengue. Serotyping of DENV was not performed, and participants were not queried for prior history of dengue infection. These factors are known to influence the severity of dengue. Seropositivity against SARS-CoV-2 or antibodies against the spike protein of SARS-CoV-2 were not measured. These can be incorporated in future studies to explore the concept of antibody-dependent enhancement of DENV in the post-COVID-19 era. The COVID-19 status of the patients was self-reported by the participants based on reverse transcription polymerase chain reaction testing. Since no tests for seropositivity for COVID-19 antibodies were done, asymptomatic or mild COVID-19 cases could not be excluded with certainty, raising the chances of underreporting of COVID-19. This being a telephonic survey, detailed clinical features and blood investigations could not be obtained. The severity of dengue was assessed in terms of the FDA definition of adverse event after immunization, and hence, specific domains of severity such as organ dysfunction or respiratory distress as highlighted in the WHO definition of severe dengue might have been missed. All participants were queried about hospitalization and the need for intravenous fluids due to hypotension, along with the lowest documented platelet count or event of bleed. In addition, the study staff recorded any additional clinical features provided by the participants, thus minimizing our chances of missing out on warning signs of dengue. The vaccinated participants had received Covishield, an adenovirus-based vaccine. The vaccination-related results of the present study hence cannot be extrapolated to other vaccines. Also, the majority of participants were health care workers, and the representation of comorbidities was meager. Individuals with comorbidities such as cardiovascular conditions and diabetes are at a higher risk of severe forms of dengue and hence should be adequately represented to understand the determinants of dengue severity. Laboratory diagnosis of dengue was made based on the serum positivity of the NS1 antigen or IgM antibodies against DENV. The association between COVID-19 and dengue was determined for dengue cases diagnosed by either of the methods, and subgroup association based only on NS1 or IgM was not performed. Because of the small number of cases with severe forms of COVID-19 and severe forms of dengue, the effect of the severity of COVID-19 on the severity of dengue could not be evaluated.^3^ The effect of other respiratory infections such as influenza and that of influenza vaccination were not explored. These limitations can be addressed in future research. Again, this being an exploratory analysis, dengue statistics were not the primary outcomes of the original two studies, and hence the current study might not be powered enough to comment upon the dengue estimates.

COVID-19 enhanced the risk of development of dengue in 2021 by 2-fold. The risk was elevated further in individuals receiving the COVID-19 vaccine after recovering from COVID-19. The severity of dengue might also be modulated by COVID-19 of the past. The possible mechanisms of immunomodulation including but not limited to T-cell suppression and antibody-dependent enhancement of dengue by SARS-CoV-2 or anti-spike antibodies should be explored. Long-term effects of COVID-19 or COVID-19 vaccines on disease course of other viral illnesses should be investigated. Larger population-level studies with better representation of dengue spectrum and comorbidities and adequate enrollment of unvaccinated people are needed to understand the patterns of modulation of dengue by COVID-19, COVID-19 vaccination, and timing of vaccination with respect to COVID-19.

## Data Availability

All data produced in the present study are available upon reasonable request made to the corresponding author, as per institutional and national legal norms and procedures.

## Supplemental files

10.4269/ajtmh.23-0418 Supplemental Materials
