# Supplementary material for: Did COVID-19 or COVID-19 Vaccines Influence the Patterns of Dengue in 2021? An Exploratory Analysis of Two Observational Studies from North India
Source: Am J Trop Med Hyg. 2023 Oct 30;109(6):1290–7. doi: 10.4269/ajtmh.23-0418 (PMC10793059; doi:10.4269/ajtmh.23-0418)
Supplement: Supplemental Materials [file tpmd230418.SD1.pdf]

	OR (CI)	P-value
**VNC (Reference)**		
CNV	4.3 (0.85-21.5)	0.07
CAV	2 (0.79-5.2)	0.14
NVNC	1.1 (0.1-11.1)	0.95
VAC	1.7 (0.65-4.3)	0.28
**NVNC (Reference)**		
CNV	4 (0.26-60.3)	0.32
CAV	1.9 (0.18-20.4)	0.59
VNC	0.93 (0.09-9.7)	0.95
VAC	1.6 (0.15-16.94)	0.71

Supplementary Table 1: **Effect estimates of dengue severity considering the categorization of individuals into five groups (Categorization B). Similar results were obtained with Categorization A.**

OR: Odds ratio, CAV: CovidAfterVaccine, CI: confidence interval, CNV: CovidNoVaccine, NVNC: NoVaccineNoCovid, VAC: VaccineAfterCovid, VNC: VaccineNoCovid
